# Medical and Surgical Treatment for Medication‐Induced Tremor: Case Report and Systematic Review

**DOI:** 10.1002/mdc3.13463

**Published:** 2022-05-24

**Authors:** Wardell E. Amerika, Saskia van der Gaag, Arne Mosch, Niels A. van der Gaag, Carel F.E. Hoffmann, Rodi Zutt, Johan Marinus, Maria Fiorella Contarino

**Affiliations:** ^1^ Department of Neurology Haga Teaching Hospital The Hague The Netherlands; ^2^ Department of Neurology Leiden University Medical Center Leiden The Netherlands

**Keywords:** deep brain stimulation, thalamus, medical treatment, medication‐induced tremor, tardive tremor

## Abstract

**Objective:**

To present a case of refractory medication‐induced tremor successfully treated with deep brain stimulation (DBS) of the thalamic ventral intermediate nucleus (Vim) and to propose a medical and surgical treatment algorithm based on a systematical review of the literature.

**Methods:**

Patient data were retrospectively collected. A systematic search was performed in PubMed, Embase, and Cochrane Library. Subjective and objective data were pooled for analysis by classifying them into 5 predefined categories(no, minimal, moderate, good, and excellent effects).

**Results:**

The patient presented with lithium‐induced bilateral progressive hand tremor lasting 25 years. After DBS, he reported excellent tremor suppression until the last follow‐up (36 months after Vim‐DBS). For the review, 34 of 140 studies were included and evaluated (178 unique subjects, 31 different treatments). A good‐to‐excellent tremor suppression (50%–100%) in at least 50% of subjects was achieved using propranolol (12 studies, 50% of 56 subjects), tetrabenazine (5 studies, 51% of 13 subjects), and metoprolol (4 studies, 75% of 8 subjects). The effect of benztropine and diphenhydramine was none or only minimal to moderate (up to 50% improvement; both: 3 studies, 50% of 4 patients). One article reported minimal‐to‐moderate effectiveness after DBS of the ventral oral posterior nucleus of the thalamus. Methods were highly heterogeneous. All studies scored grade III or IV quality of evidence, which was insufficient for recommendations (level U).

**Conclusion:**

Treatment decision making should be performed on a case‐by‐case basis considering the low level of evidence, and we propose a practically oriented treatment algorithm. Propranolol, tetrabenazine, and metoprolol might be effective. For selected and refractory cases, DBS might be considered.

Several movement disorders can occur as adverse effects of medications. Medication‐induced symptoms, including tremor, can manifest immediately after starting or stopping medication or with a delay of weeks, months, or even years. The latter are named “tardive.” Medications strongly associated with tardive syndrome(s) include neuroleptics, antiemetics, and drugs for dizziness (Table S1 in Appendix S1).^1^ The use of neuroleptics is steadily increasing internationally,^2^ bringing neurologists and psychiatrists to often encounter their adverse effects in patients. The incidence of tardive tremor in patients using neuroleptics is approximately 2%.^3^ Tardive tremor typically exhibits a coarse amplitude, has a frequency between 3 and 6 Hz, and may involve the upper and lower limbs, head, and face. It is most severe during posture, but also occurs in rest and during movement.^4^

Treatment of medication‐induced syndromes can be challenging as little evidence is available and results are conflicting. Medication‐induced tremor might respond to some tremor‐suppressing medications, but the results are often insufficient. Thalamic deep brain stimulation (DBS) is effective in the treatment of different kinds of medication‐resistant tremor.^5^, ^6^, ^7^, ^8^, ^9^, ^10^ DBS of other targets (subthalamic nucleus and globus pallidus internus) is also safe and effective in the treatment of tardive dystonia and dyskinesias.^11^, ^12^

Here, we report a severe case of lithium‐induced tremor successfully treated with bilateral DBS in the ventral intermediate nucleus (Vim). Furthermore, we systematically review the effectiveness of medical and surgical treatments in suppressing medication‐induced tremor. Finally, we propose a treatment paradigm to aid clinical decision making.

## Patients and Methods

Data concerning the case report were retrospectively collected from the patient's electronic health record. The systematic review was performed in accordance with the Preferred Reporting Items for Systematic Reviews and Meta‐Analyses guidelines.^13^

### Search Strategy

A comprehensive literature search was performed in the electronic databases PubMed (Legacy version), Embase, and the Cochrane Library up to December 10, 2019. The following search terms were used: “tardive tremor,” “medication‐induced tremor,” “medication induced tremor,” “drug‐induced tremor,” “drug induced tremor.” The limits were set to “English language” and “human” (File S2 in Appendix S1). The reference lists of retrieved studies were also reviewed. All articles retrieved using this search strategy (“potential records”) were manually screened for duplicates, which were subsequently removed from the list. The remaining articles were screened by 2 authors based on the title and abstract. Disagreements were solved by consensus.

The principal author further selected eligible studies according to inclusion and exclusion criteria by reading the full text.

### Eligibility Criteria

Studies were eligible if they met all of the following inclusion criteria: (1) 1 or more patients with medication‐induced tremor, (2) reported effectiveness of medical treatment using either subjective (qualitative scales or narrative improvement reported by the patient or examiner) or objective measurements (rating scales or measurement of tremor amplitude or frequency), and (3) English language.

Review articles, conference abstracts, and animal studies were excluded. No restrictions based on sex or ethnicity were applied.

### Risk‐of‐Bias Analysis

All included studies were subjected to a risk‐of‐bias analysis at the study level. The risk‐of‐bias assessment criteria (Table S2 in Appendix S1) were adapted from Marinus et al.^14^ A total of 2 authors reviewed the risk of bias (S.v.d.G., W.E.A.). Disagreements regarding classification were resolved by consensus.

### Data Extraction

The following data were extracted: (1) publication information, (2) tremor‐inducing drug, (3) treatment arms, (4) study design, (5) sample size, and (6) effect of each described medication. In studies involving multiple patients with different tremor types, only data from patients with medication‐induced tremors were extracted. Individual subjects within studies who were treated with multiple tremor‐reducing drugs simultaneously were excluded from the analysis. If multiple dosages of the same drug were tested, the most effective dosage was used in the analysis.

### Conversion of Tremor Improvement Rate to Intervention Effect

#### Tremor Improvement Rate: Analysis of Qualitative Data

The tremor improvement rate on qualitative data was categorized as: 0% if a treatment effect was reported as “worsening/deterioration of tremor,” “no improvement,” or “failed to resolve problem”; 12.5% (middle value of the category of improvement 0%–25% [see Calculation of Intervention Effect]) if reported as “mild,” “slight,” “some” or “modest/marginal improvement”; 37.5% if reported as “tremor improved/reduced,” “moderate improvement,” or “partially effective”; 62.5% if reported as “marked,” “good,” “much” or “clear” improvement, or “(well) controlled tremor” and “tremor not disturbing”; 87.5% if reported as “excellent,” “substantial,” “significant,” “dramatic” improvement, “very much improved,” “almost” or “complete” tremor control, “sharp reduction” “tremor remission/resolution,” “mild residual tremor,” “diminished tremor.” When a reasonable interpretation of effect was impossible because of a lack of information, the effect was considered “undetermined” and left out of subsequent calculations.

#### Tremor Improvement Rate: Analysis of Outcomes on Quantitative Data

Results were reported as a percentage of improvement on the used scale. When change in tremor was reported on a qualitative scale, we calculated the amount of improvement as a percentage.

#### Tremor Improvement Rate: Analysis of Outcomes in Case of Mixed Data

When an article applied multiple methods of tremor analysis, we pooled the results if these were consistent among each other. We considered results consistent if they did not differ by more than 1 category of overall improvement. Outcome measures were weighted equally.

#### Calculation of Intervention Effect

After averaging the tremor improvement rates of all subjects in a study, we calculated the intervention effect. We categorized the tremor improvement rate in 5 categories as follows: 0% effect was categorized as “no effect,” up to 25% improvement as “minimal effect,” 25% to 50% improvement as “moderate effect,” 50% to 75% improvement as “good effect,” and 75% to 100% improvement as “excellent effect.”

### Quality of Evidence

Articles were assessed based on the classification of evidence of the American Academy of Neurology (AAN).^15^, ^16^ Recommendations were linked to the level of evidence.^17^ Two authors reviewed the levels of evidence (W.E.A., M.F.C.). Disagreements regarding classification were resolved by consensus.

## Results

### Case Report

A 72‐year‐old man with a history of bipolar disorder had been treated with lithium for 30 years (up to 800 mg daily), and during the past 25 years, he had developed a progressive bilateral tremor of the hands. Other than an overactive bladder, his medical history was unremarkable. There was no family history of movement disorders or other neurological diseases. At the time of presentation, his medication included fluoxetine (20 mg daily), lithium (400 mg daily), mirabegron (50 mg daily), and desmopressin (120 μg daily). In the past, he had temporarily been treated with valproic acid for his bipolar disorder.

A temporary reduction of lithium dosage resulted in a clear improvement of the tremor, but unfortunately induced a relapse into a depression unresponsive to other treatments, and hence lithium was reinstituted. Tremor did not respond to levodopa/carbidopa (500 mg daily). Tetrabenazine and propranolol were contraindicated because of depressive symptoms and the use of fluoxetine.

Neurological examination revealed severe rest, postural, and kinetic tremor in both arms and minimal rest tremor and moderate postural tremor in both legs, predominant on the right side of the body. Gait was normal. Tendon reflexes were symmetrically brisk, with indifferent plantar reflexes. There was no evidence of rigidity, bradykinesia, or ataxia.

Blood testing revealed a therapeutic lithium concentration and no significant abnormalities. Brain magnetic resonance imaging (MRI) was normal. Neuropsychological testing showed no cognitive decline. Psychiatric symptoms were stable.

Based on the clinical presentation (progressive rest, postural, and kinetic tremor) and history of long‐term use of lithium, with clear improvement during temporary reduction of the dosage, a diagnosis of tardive tremor was made. There were no contraindications for DBS. Patient underwent awake bilateral Vim DBS surgery based on stereotactic MRI with Leksell frame as previously described, with intraoperative microelectrode recording and test stimulation.^18^ The target was determined bilaterally 16 mm lateral and 4 mm posterior to mid‐commissural point, at the depth of the anterior commissure ‐ posterior commissure (AC‐PC) line. The central track was chosen for definitive lead implantation (lead model 3389; Medtronic, Minneapolis, MN) on the right side and the posterior track on the left side.

Because of the stun effect of leads placement, tremor severity was markedly reduced bilaterally during 4 days following surgery and gradually returned to the preoperative situation within 9 days, when the Essential Tremor Rating Assessment Scale (TETRAS) part A score was 28. Drinking from a glass with the right hand was not possible without spilling. Writing and drawing Archimedes' spiral was severely affected bilaterally (Video 1). At the follow‐up visit 9 days after surgery, a standard impedance check and monopolar contact review were performed. With monopolar stimulation at the deepest electrode in the left hemisphere (3.0 mA, 60 μs, 180 Hz) and at the second‐deepest electrode in the right hemisphere (2.2 mA, 60 μs, 180 Hz), the patient experienced an immediate improvement of tremor, with residual minimal resting tremor of the left hand and leg upon distraction and mild proximal postural and kinetic tremor. Accordingly, tasks such as drinking from a glass, writing, and drawing Archimedes' spiral were significantly improved (TETRAS part A score 4; Video 2). There were no stimulation‐induced adverse effects at the effective stimulation settings.

**Video 1:** Ten days after ventral intermediate deep brain stimulation surgery, with stimulation still switched OFF. The patient presents with severe rest, postural, and kinetic tremor involving the upper and lower limbs (right more than left). No re‐emerging tremor phenomenon. Drinking from a cup with the right hand is not possible without spilling. Writing and drawing of Archimedes' spiral is severely affected.

**Video 2:** Ten days after ventral intermediate deep brain stimulation surgery, immediately after monopolar contact review, with stimulation turned ON (right: second‐deepest contact monopolar, 2.2 mA; left: deepest contact monopolar, 3.0 mA; both with 130 Hz frequency and 60 μs pulse width). Tremor suppression, with residual minimal resting tremor of the left upon distraction, and mild proximal postural and kinetic tremor. Drinking from a glass, writing, and drawing of Archimedes' spiral are significantly improved.

Routine follow‐up neuropsychological testing 6 months after surgery revealed no change. At the last follow‐up, 36 months after surgery, tremor remained suppressed to the patient's satisfaction (Video 3). His psychiatric status remained unchanged. The chronic selected settings were bilateral monopolar stimulation using the deepest electrode in the left hemisphere (3.2 mA, 60 μs, 180 Hz) and the deepest electrode in the right hemisphere (2.5 mA, 60 μs, 180 Hz).

**Video 3:** Three years after ventral intermediate deep brain stimulation. Tremor suppression substantially unchanged with minimal residual kinetic tremor.

### Literature Review

**FIG. 1:**
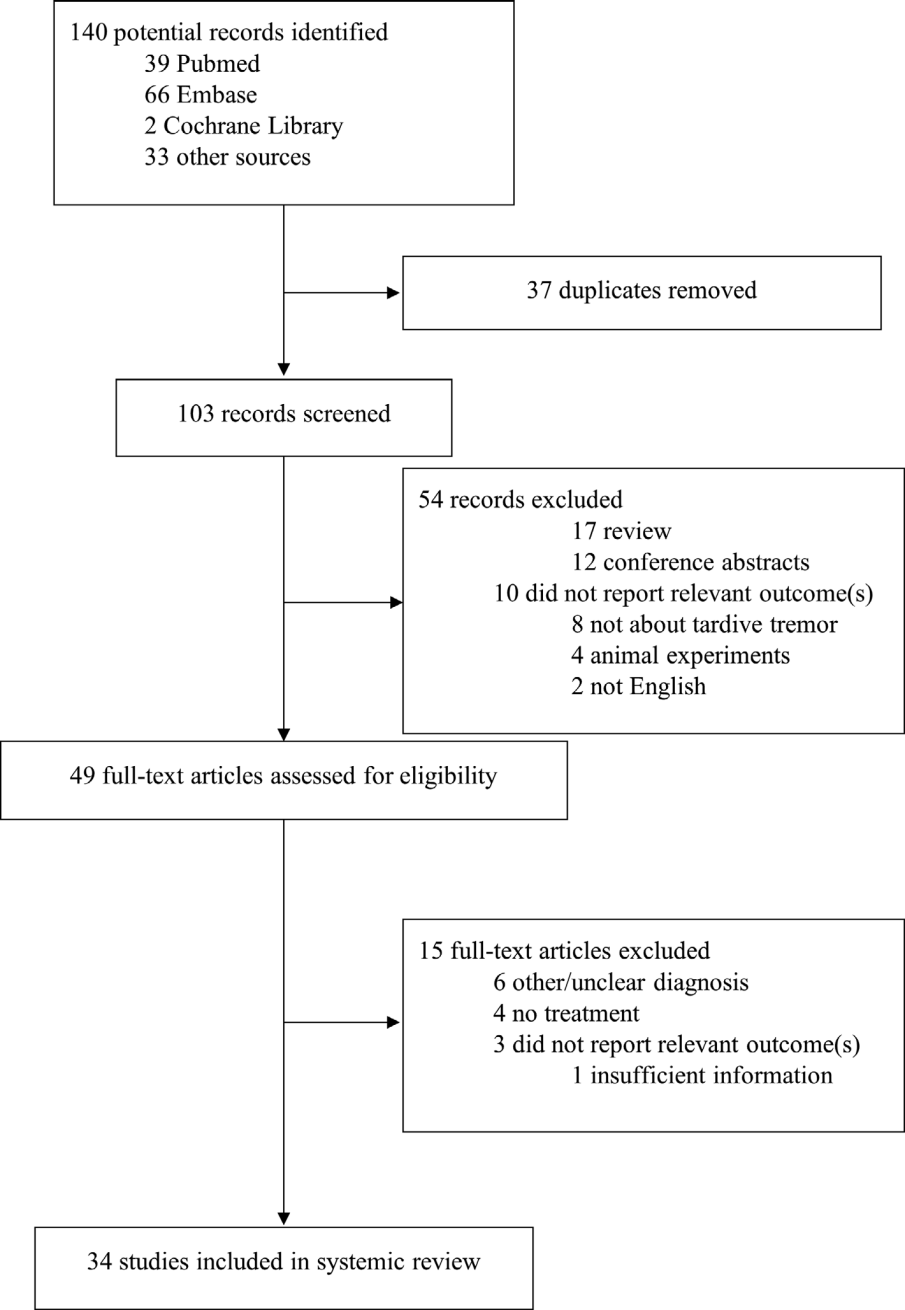
Preferred Reporting Items for Systematic Reviews and Meta‐Analyses flowchart of study selection in systematic review.

The literature search identified 140 records of which 34 studies met the inclusion criteria and were analyzed (Fig. 1).

#### Study Characteristics and Quality

The characteristics of the included studies are shown in Tables S3 and S4 in Appendix S1. All studies were case reports or case series (30) or randomized controlled trials (RCTs; 4) published between 1973 and 2015. In 228 interventions, 30 different drugs and 1 stereotactic treatment were investigated.^19^ There were 178 unique subjects. Some of the subjects underwent several measurements with different medications. According to the AAN level of evidence, 31 articles had a quality grade IV, and 3 articles had a quality grade III. A total of 25 articles used qualitative outcome measures to describe tremor improvement, including patient or examiner experience (20 studies), assessment of spiral drawing (3), handwriting (2), and finger–nose testing (1). A total of 10 studies used quantitative methods, including accelerometer recordings (3); the tremor subscale of the Simpson‐Angus Scale (2)^20^; the Subjective Clinical Improvement Impression Scale, which is a modification of the Clinical Global Impressions Scale (2)^21^, ^22^; the Abnormal Involuntary Motor Scale (1)^23^; the Clinical Global Impression of Change Scale (1)^24^; TETRAS (1)^25^; and other various tremor rating scales (4 studies).^24^, ^26^

#### Patient and Tremor Descriptions

Clinical data reporting (medication, dosage, age, sex, tremor location, type, and frequency) was incomplete in many studies. From available data, ages ranged from 18 to 79 years, and there were at least 54 men and 47 women. Lithium was the most common tremor‐inducing drug, reported as a single causative agent in 102 subjects (45% of total). Tremor location was mostly reported in the upper extremities with or without additional locations such as head or lower extremities. The trunk was involved in only 1 case.^27^ Of 21 articles reporting tremor type, 17 described a combination of postural (16 articles), resting (13), kinetic (5), intention tremor (5), and unspecified action tremor (3). A total of 4 articles reported a single tremor type: postural (3) or unspecified action tremor (1). Tremor frequency (11 articles) ranged between 3 and 10 Hz.

#### Risk of Bias

With regard to risk of bias, 1 study was scored as a high‐quality study, 8 studies as medium quality, and 25 studies as poor quality (Table S5 in Appendix S1).

#### Intervention Effect per Treatment

**FIG. 2:**
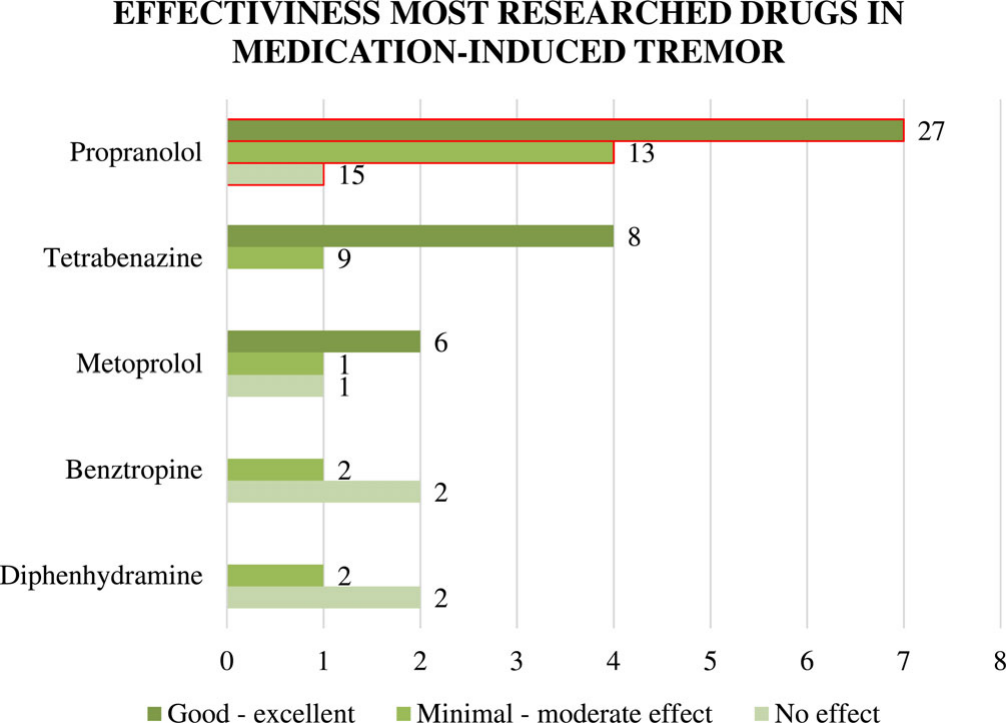
Effectiveness of most investigated drugs, for which at least 3 articles were available. The investigated drugs are shown on the y‐axis, and the amount of studies pertaining a particular result are shown on the x‐axis. Effects are divided in 3 groups: good to excellent, minimal to moderate, and no effect. The numbers at the end of each bar represent the number of subjects. A red border indicates the inclusion of 1or more level III quality studies.

Propranolol, tetrabenazine, metoprolol, benztropine, and diphenhydramine were tested in at least 3 articles; another 25 medications and DBS were tested in fewer than 3 articles, with only 1 medication (oxprenolol) studied in a larger study population.^28^ The effect of treatments studied in at least 3 articles are shown in Figure 2. Propranolol up to 240 mg daily was investigated in 12 articles (including 3 grade III quality studies), with a total of 56 subjects.Among the medications tested in fewer than 3 articles, the following 3 medications were tested in at least 1 class III study: sotalol, practolol, and atenolol. The class III studies on sotalol 0.5 mg/kg showed a good‐to‐excellent intervention effect and the study on atenolol showed minimal‐to‐moderate effect, whereas the study on practolol 120 mg daily showed no effect. In this study, sotalol and atenolol were injected intravenously during a 4‐minute period in weekly intervals. All other treatments examined in fewer than 3 articles were studied in class IV studies only.

#### Intervention Effect per Treatment Class

We grouped all interventions per treatment class (Table 1). The largest number of studies, as well as the only class III evidence studies, were performed with β‐blockers (25 studies, 146 subjects). With respect to surgery, only 1 case report was found on ventral oral posterior nucleus of the thalamus (Vop) DBS with a minimal‐to‐moderate effect.^19^

**TABLE 1:** *Overview of investigated drugs with their effectiveness*, *studied in 227 interventions with 178 unique subjects in 34 articles*

Class	Drug	No effect	Minimal to moderate	Good to excellent	Total
β‐blockers	Total	3^a^	8^a^	14^a^	25^a^
	Arotinolol			1	1
	Atenolol		1^a^	1	2^a^
	Metoprolol	1	1	2	4
	Nadolol			2	2
	Oxprenolol		2		2
	Practolol	1^a^			1^a^
	Propranolol	1^a^	4^a^	7^a^	12^a^
	Sotalol			1^a^	1^a^
Anticholinergics	Total	3	3		6
	Amantadine		1		1
	Benztropine	2	1		3
	Ethopropazine		1		1
	Trihexyphenidyl	1			1
Monoamine reuptake inhibitors	Total	1	1	4	6
	Reserpine	1			1
	Tetrabenazine		1	4	5
GABAergics	Total	1	2	2	5
	Clonazepam	1	1		2
	Pregabalin			1	1
	Primidone		1	1	2
Antihistaminergics	Total	2	2		4
	Cryptoheptadine		1		1
	Diphenhydramine	2	1		3
Dopaminergic drugs	Total	2	1		3
	Bromocriptine	1	1		2
	Carbidopa/levodopa	1			1
Vitamins	Total	1		2	3
	Inositol	1		1	2
	Vitamin B6			1	1
Dopamine antagonists	Total		1	1	2
	Clozapine			1	1
	Haloperidol		1		1
Fatty acids	Linoleic acid	1		1	2
Carbonic anhydrase inhibitors	Acetazolamide			1	1
Cholinesterase inhibitors	Donepezil			1	1
Electrolytes	Potassium			1	1
NaSSA	Mirtazapine	1			1
DBS	Vop‐DBS		1		1

Numbers indicate the number of articles stating the effect.

^a^ One or more level III studies.

NaSSA, noradrenergic and specific serotonergic antidepressant; DBS, deep brain stimulation; GABA, gamma‐aminobutyric acid; Vop, ventral oral posterior nucleus of the thalamus.

#### Level of Recommendation

There were no class I or II quality‐of‐evidence studies. Although 4 studies were RCTs, these did not meet all the criteria for classification of AAN class I or II. As such, the effect of all investigated treatments is considered unproven (level U recommendation: insufficient data to support or refute use of a particular treatment).

#### Flowchart

**FIG. 3:**
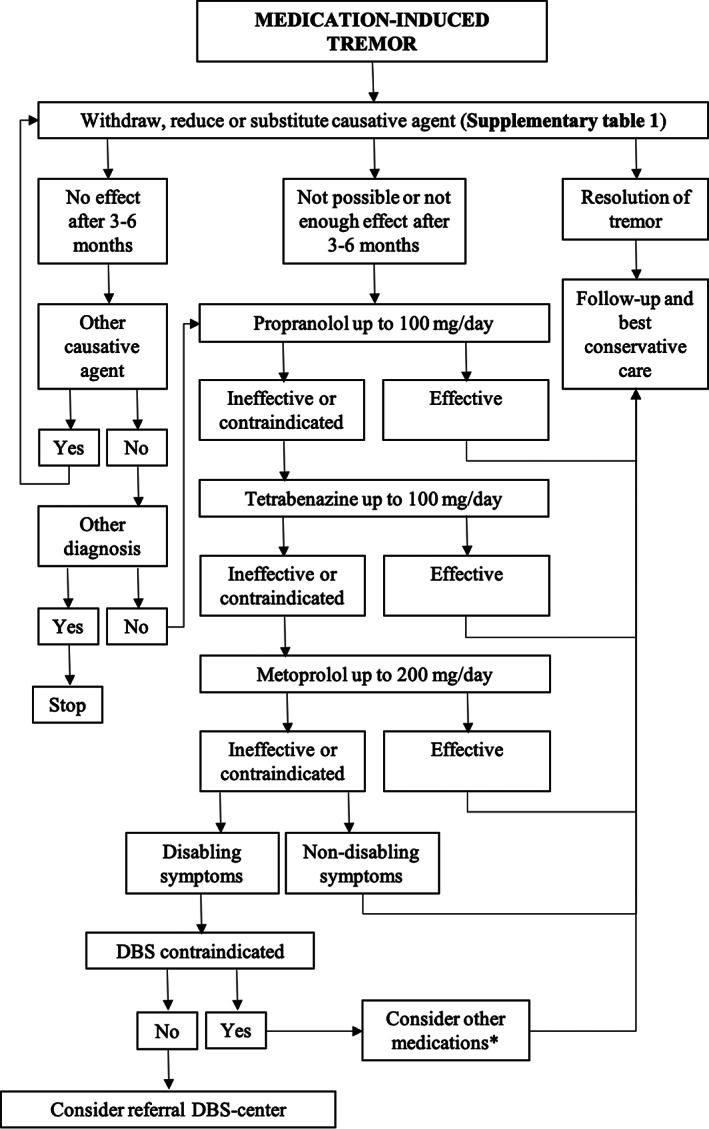
Suggested flowchart for the treatment of medication‐induced tremor. The steps suggesting deep brain stimulation (DBS) or use of other medications are marked as “consider to” because of the scanty of available evidence. *Nadolol, acetazolamide, donepezil, potassium, pregabalin, primidone, sotalol, or vitamin B6.

To aid clinical decision making, we propose here a paradigm for the management of bothersome medication‐induced tremor based on the aforementioned reported results (Fig. 3). The first proposed step is to attempt sequential withdrawal, reduction, or substitution of the most likely causative agents for a sufficient period (eg, 3–6 months), similar to what is suggested for other tardive syndromes.^29^ In 1 prospective cohort study, the response rate to the withdrawal of dopamine modulators was as low as 1% to 20%, with a remission rate of only 2%,^30^ whereas a post hoc analysis found a higher improvement rate after discontinuation of neuroleptics in patients with tardive dyskinesia (81% in patients aged younger than 54.5 years and 46% in older patients).^31^ Despite insufficient evidence for the efficacy of medication withdrawal (also for other medication‐induced syndromes), this appears to be a logical first step in clinical practice.

If medication withdrawal is not possible or the tremor persists, we advise starting medication. In the flowchart, priority is given to the medications for which more evidence for good‐to excellent effect is available. The first choice is thus reserved to treatment with propranolol up to 100 mg daily (US Food and Drug Administration [FDA]–suggested maximum dosage: 240 mg daily for essential tremor). As a second choice, tetrabenazine could be considered up to 100 mg/day. The FDA advises not to increase the dosage above 50 mg/day before cytochrome P450 2D6 genotyping, as poor metabolizers are at an increased risk of overdosing.

If these treatments prove unsuccessful or produce adverse effects, another β‐blocker such as metoprolol up to 200 mg/day may be useful as a next step.

If no medical treatment is successful and symptoms are disabling, DBS could be considered based on the large experience with tremor of other origins and the 2 available cases (including the 1 described here) reporting encouraging results on medication‐induced tremor. However, because this treatment is still supported by scanty evidence, this step in the flowchart is marked as “consider to” to underline that the choice should be made on clinical grounds on a case‐by‐case basis. Although there is ample experience with thalamotomy to treat tremor of other origins, we did not mention this explicitly in the flowchart because of the lack of reported cases with medication‐induced tremor. Also, thalamotomy is currently advised as a unilateral treatment, whereas medication‐induced tremor is most often bilateral.If stereotactic surgery is contraindicated, other second‐line medications for which less evidence is available could still be tried. In this context, preference could be given to oxprenolol, a medication that showed minimal‐to‐moderate effect in 2 articles that included a large number of subjects.^28^, ^33^ Also for this step, given the low quality of evidence, caution should be used.

## Discussion

Treatment of refractory medication‐induced tremor is challenging, and (international) guidelines are not available. We report the successful long‐term effect of thalamus DBS (Vim) in a patient with a severe treatment‐resistant medication‐induced tremor, which had been reported only in another case previously (Vop). Furthermore, our systematic review showed that the existing literature is highly heterogeneous. Studies were scarce and mostly limited to case reports or small case series. Therefore, the quality of studies, both in terms of quality of evidence as well as risk of bias, was low; with regard to quality of evidence, there were no high‐quality controlled trials, and the vast majority of studies only met criteria for class IV (and a few class III). With regard to the risk of bias, there was only 1 high‐quality study, few medium‐quality studies, and many poor‐quality studies. This indicates a high risk of bias within the included studies.

The lack of high‐quality evidence on the treatment of medication‐induced tremor is most likely multifactorial. One possible reason is the relative rarity of the disorder (2.4% among people using antipsychotics)^3^ partly because of the underreporting by patients and unawareness of physicians.^34^ Other factors are the heterogeneity of the presentation, cause, and reaction to treatment and the lack of biomarkers and consensus criteria to make a definite diagnosis, which make it difficult to gather large homogeneous cohorts.

Notwithstanding these limitations, the available literature suggests that propranolol, tetrabenazine, and metoprolol may be effective treatments, where propranolol has been studied most extensively.

In the proposed treatment flowchart (Fig. 3), we propose to consider sequential withdrawal of suspect medication as a first step. The persistence of diagnosed medication‐induced movement disorders for months or even years after withdrawal of the suspected medication has been previously reported.^35^ The quite low^30^ or incomplete^31^ response rates reported in some studies could be caused by a (long‐term) alteration in the receptor profile or maladaptive plasticity changes, but could also indicate an insufficient follow‐up, progression of a preexisting unnoticed movement disorder, or a latent condition (eg, Parkinson's disease, essential tremor) exposed by the use of the suspected drug.

Medication withdrawal is not always possible on clinical grounds and should always be closely supervised to monitor the underlying disorder, especially in case of psychiatric disorders, ideally in the context of a multidisciplinary team.

β‐blockers in general were the most studied drug category with the highest class of evidence and showed an overall good effect on medication‐induced tremor. Explaining the effectiveness of these treatments on a structural level is difficult, as the pathophysiology of medication‐induced tremor is poorly understood.^36^ The effectiveness of β‐blockers is also supported by the clinical experience with these agents in other types of tremors, such as essential and enhanced physiological tremors. Multiple studies have argued that the tremorolytic action of β‐blockers is peripherally mediated, possibly at the level of the β‐2 adrenergic receptors of skeletal muscle spindles.^37^, ^38^, ^39^ This would explain the nonspecific effect of β‐blockers on different kinds of tremor. Although results indicate mainly good‐to‐excellent effect in monoamine reuptake inhibitors, this was driven almost exclusively by studies with tetrabenazine. Tetrabenazine is a benzoquinolizine pharmacore that selectively depletes vesicular monoamines through inhibition of vesicular monoamine transporter type 2 (VMAT2) and improves hyperkinetic syndromes.^40^ Because tardive syndromes are thought to be caused by dopamine hypersensitivity, reducing postsynaptic dopamine receptor stimulation by inhibition of VMAT2 may also improve tardive syndromes.^41^, ^42^ For tetrabenazine there is level C recommendation that it is effective for the treatment of tardive dyskinesia,^12^ suggesting that it could be effective in tardive tremor as well.

It is also important to notice that new compounds of the family of vesicular monoamine transporter 2 inhibitors, namely, deutetrabenazine and valbenazine, have recently entered the market. Both are shown to be safe and are FDA approved.^43^, ^44^, ^45^ Deutetrabenazine seems to be safer than tetrabenazine in the treatment of Huntington's disease.^46^ Although there are currently no reports for the treatment of tardive tremor, this might be a promising option in the future.^47^ Studies on anticholinergics and antihistaminic drugs produced disappointing results with either minimal‐to‐moderate or no effect. Although anticholinergics are commonly used in the treatment of tardive syndromes, the available evidence does not confirm their efficacy.^48^, ^49^

Results on a number of drugs studied in fewer than 3 articles (acetazolamide, arotinolol, donepezil, potassium, pregabalin, primidone, sotalol, and vitamin B6) show good‐to‐excellent effect, indicating that these drugs may be candidates for future research as well. Importantly, all of the aforementioned drugs have a specific range of adverse effects, which can be more common in certain categories of patients. For example, β‐blockers are contraindicated in patients with asthma and should be used with caution in patients under treatment for cardiovascular pathologies, tetrabenazine is contraindicated in patients with depressive symptoms or a history of suicide attempts, and anticholinergics have a high incidence of adverse effects (eg, cognitive problems, dizziness, constipation), especially in older individuals.

DBS treatment may be effective in medication‐induced tremor: our literature search revealed 1 case with minimal‐to‐moderate effectiveness of Vop‐DBS, and our own case showed a good‐to‐excellent effect of Vim‐DBS. In another case, Vim‐DBS showed a good‐to‐excellent response on a possible tardive tremor. However, this study was excluded from this review because the authors could not rule out essential tremor as an alternative diagnosis because of a positive family history for tremor and the responsiveness of tremor to alcohol.^50^

Although evidence for the effectiveness of DBS on medication‐induced tremor is very scarce and therefore definite conclusions cannot be drawn, positive effects of thalamic DBS have been shown in other forms of tremor^5^, ^51^ and in other tardive syndromes (with different targets).^11^, ^12^

However, the risks related to DBS surgery need to be considered before indicating this invasive therapy.^52^ Furthermore, DBS for movement disorders is contraindicated for subjects with active psychiatric conditions. Subjects with a history of psychiatric episodes are considered to be at risk of symptom recurrence after surgery as a result of stressors around the surgical procedure and possibly also attributed to direct stimulation effects. Nevertheless, this has not been frequently reported in patients with tardive syndromes who had a stable control of the psychiatric symptoms at the time DBS surgery.^32^ Similarly, no psychiatric adverse effects or deterioration or previous psychiatric conditions were observed in our patient, who was under stable symptom control at the time of surgery. However, as the perspective of these positive effects and possible risks are valued differently by every patient, decision making seems best to be performed on a case‐by‐case basis and after extensive discussion with the patients and their caregivers.Given the heterogeneity of this class of patients, the choice of both medical and surgical treatment strategies should also be accurately weighed against possible individual contraindications and the subjective risk of specific medication‐induced adverse effects, thus the diagram should be evaluated case by case.

Our study has several limitations. First, there was a large variety of medications reported to induce tremor as well as a large variety of treatments investigated with heterogeneous methods. Most of the studies were case reports and case series, which could generate a publication bias, perhaps leading to an overestimation of the effects. Unfortunately, because the quality of the included studies was low, lacking large randomized controlled clinical trials and comparative trials and often presenting incomplete clinical data, all reported effects remain unproven (level U recommendation), which makes it difficult to draw definite conclusions. Furthermore, reports were not always accurate, for example, a clear distinction between tardive tremor and other forms of medication‐induced tremor was often missing. However, we do not expect that treatment of medication‐induced tremor in general would differ substantially from treatment of tardive tremor.

Because of the low quality of the majority of articles included, there was insufficient information available to properly verify the originally reported diagnoses of medication‐induced tremor. Therefore, we accepted the original authors' diagnoses, possibly leading to bias in our review. Another limitation is the necessity of converting subjective into objective data, possibly inducing errors. Also, because of the scarcity of studies and the lack of identification of the causative agents, we could not subcategorize the results, leading to generalized conclusions. Lastly, we used the number of articles that examined the intervention as a way of determining the power of each intervention effect because the number of different sources can be important to establish the credibility of the results. As most articles included only a few participants, classifying treatment effects based on the number of patients tested instead would have resulted in similar results, with oxprenolol as the only notable exception, as mentioned previously.

Notwithstanding the limitations, we believe our results do have merit. First, this is the first effort to review the medical and surgical treatment options for medication‐induced tremor. With our categorization method, we managed to pool results from a heterogeneous group of studies. Despite the lack of high‐quality evidence, our data clearly identify at least 3 medications that appear to have good‐to‐excellent effectiveness (propranolol) or a trend toward good‐to‐excellent effectiveness (tetrabenazine and metoprolol). Moreover, we identified other medications that are mostly proven not useful and could be avoided in clinical practice. Based on this knowledge, we created a treatment algorithm that may serve as a starting point to aid clinical decision making.

In conclusion, our review identified a clear knowledge gap, which could be used a starting point to guide future research into medication‐induced tremor. In particular, high‐quality studies are not only needed for the drugs that have had promising results in preliminary reports but also the new medications that have recently entered the market, such as deutetrabenazine, valbenazine, or perampanel. Finally, the successful treatment of our patient with a severe, refractory medication‐induced tremor with Vim‐DBS should encourage clinicians to consider referral of similar patients to a DBS center to explore the possibility of stereotactic treatment.

## Author Roles

(1) Research Project: A. Conception, B. Organization, C. Execution; (2) Statistical Analysis: A. Design, B. Execution, C. Review and Critique; (3) Manuscript: A. Writing of the First Draft, B. Review and Critique.

W.E.A.: 1C, 2A, 2B, 3A

S.v.d.G.: 1C, 2C, 3B

A.M.: 1C, 2C, 3B

N.A.v.d.G.: 1C, 2C, 3B

C.F.E.H.: 1C, 2C, 3B

R.Z.: 1C, 2C, 3B

J.M.: 1C, 2A, 2C, 3B

M.F.C.: 1A, 1B, 2C, 3B

## Disclosures

### Ethical Compliance Statement

Patient's written informed consent for publication of his data and video was obtained. The authors confirm that the approval of an institutional review board was not required for this work. We confirm that we have read the Journal's position on issues involved in ethical publication and affirm that this work is consistent with those guidelines.

### Funding Sources and Conflicts of Interest

The authors declare no funding sources or conflicts of interest.

### Financial Disclosures for the Previous 12 Months

W.E.A. received speaking fees from Teva. M.F.C. participated in advisory board and received speaking fees from Boston Scientific; received unrestricted grants, participated in advisory board, and received speaking fees and consultancies from Medtronic; performed consultancy for the Center for Human Drug Research; received grants from AbbVie; and received in‐kind contribution from Global Kinetics Corporation all outside the submitted work. All fees are paid to the institution. S.v.d.G., A.M., N.v.d.G., C.F.E.H., R.Z., and J.M. report no disclosures.

## Supporting information


**Appendix S1** Supporting Information

## References

[1] Fabiani G , Pastro P , Froehner C . Parkinsonism and other movement disorders in outpatients in chronic use of cinnarizine and flunarizine. Arq Neuropsiquiatr 2004;62(3B):784–788. 15476069 10.1590/s0004-282x2004000500008

[2] Halfdanarson O, Zoega H, Aagaard L , et al. International trends in antipsychotic use: a study in 16 countries, 2005‐2014. Eur Neuropsychopharmacol 2017;27(10):1064–1076. 28755801 10.1016/j.euroneuro.2017.07.001

[3] Lee MJ, Lin PY, Chang YY, Chong MY, Lee Y . Antipsychotics‐induced tardive syndrome: a retrospective epidemiological study. Clin Neuropharmacol 2014;37(4):111–115. 24992086 10.1097/WNF.0000000000000040

[4] Stacy M , Jankovic J . Tardive tremor. Mov Disord 1992;7(1):53–57. 1348352 10.1002/mds.870070110

[5] Ramirez‐Zamora A , Okun MS . Deep brain stimulation for the treatment of uncommon tremor syndromes. Expert Rev Neurother 2016;16(8):983–997. 27228280 10.1080/14737175.2016.1194756 PMC4975099

[6] Koller WC, Lyons KE, Wilkinson SB, Pahwa R . Efficacy of unilateral deep brain stimulation of the VIM nucleus of the thalamus for essential head tremor. Mov Disord 1999;14(5):847–850. 10495050 10.1002/1531-8257(199909)14:5<847::aid-mds1021>3.0.co;2-g

[7] Rehncrona S , Johnels B, Widner H, Tornqvist AL, Hariz M, Sydow O. Long‐term efficacy of thalamic deep brain stimulation for tremor: double‐blind assessments. Mov Disord 2003;18(2):163–170. 12539209 10.1002/mds.10309

[8] Koller WC, Lyons . Long‐term safety and efficacy of unilateral deep brain stimulation of the thalamus in essential tremor. Mov Disord 2001;16(3):464–468. 11391740 10.1002/mds.1089

[9] Zesiewicz TA et al. Practice parameter: therapies for essential tremor: report of the Quality Standards Subcommittee of the American Academy of Neurology. Neurology 2005;64(12):2008–2020. 15972843 10.1212/01.WNL.0000163769.28552.CD

[10] Dallapiazza RF, Lee DJ, De Vloo P , et al. Outcomes from stereotactic surgery for essential tremor. J Neurol Neurosurg Psychiatry 2019;90(4):474–482. 30337440 10.1136/jnnp-2018-318240 PMC6581115

[11] Macerollo A , Deuschl G . Deep brain stimulation for tardive syndromes: systematic review and meta‐analysis. J Neurol Sci 2018;389:55–60. 29433807 10.1016/j.jns.2018.02.013

[12] Bhidayasiri R, Jitkritsadakul O, Friedman JH, Fahn S . Updating the recommendations for treatment of tardive syndromes: a systematic review of new evidence and practical treatment algorithm. J Neurol Sci 2018;389:67–75. 29454493 10.1016/j.jns.2018.02.010

[13] Moher D, Liberati A, Tetzlaff J, Altman DG, PRISMA Group . Preferred reporting items for systematic reviews and meta‐analyses: the PRISMA statement. J Clin Epidemiol 2009;62(10):1006–1012. 19631508 10.1016/j.jclinepi.2009.06.005

[14] Marinus J, Zhu K, Marras C, Aarsland D, van Hilten JJ . Risk factors for non‐motor symptoms in Parkinson's disease. Lancet Neurol 2018;17(6):559–568. 29699914 10.1016/S1474-4422(18)30127-3

[15] Appendix C . AAN classification of evidence for the rating of a therapeutic study. Continuum (Minneap Minn) 2015;21(4 Headache):1169. 26252602 10.1212/01.CON.0000470920.20859.fe

[16] Gross RA , Johnston KC . Levels of evidence: taking neurology to the next level. Neurology 2009;72(1):8–10. 19122025 10.1212/01.wnl.0000342200.58823.6a

[17] Yadav V, Bever C Jr, Bowen J , et al. Summary of evidence‐based guideline: complementary and alternative medicine in multiple sclerosis: report of the guideline development subcommittee of the American Academy of Neurology. Neurology 2014;82(12):1083–1092. 24663230 10.1212/WNL.0000000000000250 PMC3962995

[18] Geraedts VJ, van Ham RAP, Marinus, et al. Intraoperative test stimulation of the subthalamic nucleus aids postoperative programming of chronic stimulation settings in Parkinson's disease. Parkinsonism Relat Disord 2019;65:62–66. 31105015 10.1016/j.parkreldis.2019.05.017

[19] Milosevic L, Dallapiazza RF, Munhoz RP, Kalia SK, Popovic MR, Hutchison WD . Case studies in neuroscience: lack of inhibitory synaptic plasticity in the substantia nigra pars reticulata of a patient with lithium‐induced tremor. J Neurophysiol 2019;122(4):1367–1372. 31411948 10.1152/jn.00203.2019 PMC6843100

[20] Simpson GM , Angus JW . A rating scale for extrapyramidal side effects. Acta Psychiatr Scand Suppl 1970;212:11–19. 4917967 10.1111/j.1600-0447.1970.tb02066.x

[21] Guy W . ECDEU Assessment Manual for Psychopharmacology, Revised. Rockville, MD: National Institute of Mental Health US Dept Health, Education and Welfare publication (ADM); 1976:76–338.

[22] Miodownik C , Witztum E , Lerner V . Lithium‐induced tremor treated with vitamin B6: a preliminary case series. Int J Psychiatry Med 2002;32(1):103–108. 12075913 10.2190/DB1V-85M4-E65T-R3QA

[23] Munetz MR , Benjamin S . How to examine patients using the Abnormal Involuntary Movement Scale. 1988, Hosp Community Psychiatry;39(11):1172–1177. 10.1176/ps.39.11.1172 2906320

[24] Kertesz DP, Swartz MV, Tadger S, Plopski I, Barak Y . Tetrabenazine for tardive tremor in elderly adults: a prospective follow‐up study. Clin Neuropharmacol 2015;38(1):23–25. 25580924 10.1097/WNF.0000000000000061

[25] Elble R, Comella C, Fahn S , et al. Reliability of a new scale for essential tremor. Mov Disord 2012;27(12):1567–1569. 23032792 10.1002/mds.25162 PMC4157921

[26] Zubenko GS , Cohen BM , Lipinski JF Jr . Comparison of metoprolol and propranolol in the treatment of lithium tremor. Psychiatry Res 1984;11(2):163–164. 6584937 10.1016/0165-1781(84)90100-8

[27] Patterson RG , Couchenour RL . Trimethoprim‐sulfamethoxazole‐induced tremor in an immunocompetent patients. Pharmacotherapy 1999;19(12):1456–1458. 10600097 10.1592/phco.19.18.1456.30903

[28] Poldinger W . Therapy of extrapyramidal side effects, with particular reference to persistent dyskinesia and lithium tremor. Int Pharmacopsychiatry 1978;13(4):230–233. 32149 10.1159/000468344

[29] Morgan JC , Sethi KD . Drug‐induced tremors. Lancet Neurol 2005;4(12):866–876. 16297844 10.1016/S1474-4422(05)70250-7

[30] Glazer WM, Morgenstern H, Schooler N, Berkman. Predictors of improvement in tardive dyskinesia following discontinuation of neuroleptic medication. Br J Psychiatry 1990;157:585–592. 1983390 10.1192/bjp.157.4.585

[31] Smith JM , Baldessarini RJ . Changes in prevalence, severity, and recovery in tardive dyskinesia with age. Arch Gen Psychiatry 1980;37(12):1368–1373. 6108750 10.1001/archpsyc.1980.01780250054006

[32] Mentzel CL, Tenback DE, Tijssen MAJ, Visser‐Vandewalle VERM, van Harten PN . Efficacy and safety of deep brain stimulation in patients with medication‐induced tardive dyskinesia and/or dystonia: a systematic review. J Clin Psychiatry 2012;73(11):1434–1438. 23218160 10.4088/JCP.12r07643

[33] Brosteanu EFL , Kaiser H . Double‐blind trial with oxprenolol versus placebo in the treatment of lithium‐induced tremor. In: Kielholz P , ed. Beta‐Blockers and the Central Nervous System. Baltimore, MD: University Park Press; 1977.

[34] Tarsy D , Baldessarini RJ . Epidemiology of tardive dyskinesia: is risk declining with modern antipsychotics? Mov Disord 2006;21(5):589–598. 16532448 10.1002/mds.20823

[35] Waln O , Jankovic J . An update on tardive dyskinesia: from phenomenology to treatment. Tremor Other Hyperkinet Mov (N Y) 2013;3: tre‐03‐161‐4138‐1. 10.7916/D88P5Z71 PMC3709416 23858394

[36] Morgan JC, Kurek JA, Davis JL, Sethi KD . Insights into pathophysiology from medication‐induced tremor. Tremor Other Hyperkinet Mov (N Y) 2017;7:442. 29204312 10.7916/D8FJ2V9Q PMC5712675

[37] Abila B, Wilson JF, Marshall RW, Richens A . The tremorolytic action of beta‐adrenoceptor blockers in essential, physiological and isoprenaline‐induced tremor is mediated by beta‐adrenoceptors located in a deep peripheral compartment. Br J Clin Pharmacol 1985;20(4):369–376. 2866785 10.1111/j.1365-2125.1985.tb05079.x PMC1400891

[38] Jefferson D , Jenner P , Marsden CD . beta‐Adrenoreceptor antagonists in essential tremor. J Neurol Neurosurg Psychiatry 1979;42(10):904–909. 512665 10.1136/jnnp.42.10.904 PMC490362

[39] Leigh PN, Jefferson D, Twomey A, Marsden CD . Beta‐adrenoreceptor mechanisms in essential tremor; a double‐blind placebo controlled trial of metoprolol, sotalol and atenolol. J Neurol Neurosurg Psychiatry 1983;46(8):710–715. 6310053 10.1136/jnnp.46.8.710 PMC1027523

[40] Fasano A , Bentivoglio AR . Tetrabenazine. Expert Opin Pharmacother 2009;10(17):2883–2896. 19929707 10.1517/14656560903386292

[41] Bhidayasiri R, Fahn S, Weiner WJ , et al. Evidence‐based guideline: treatment of tardive syndromes: report of the Guideline Development Subcommittee of the American Academy of Neurology. Neurology 2013;81(5):463–469. 23897874 10.1212/WNL.0b013e31829d86b6

[42] German CL, Baladi MG, McFadden LM, Hanson GR, Fleckenstein AE . Regulation of the dopamine and vesicular monoamine transporters: pharmacological targets and implications for disease. Pharmacol Rev 2015;67(4):1005–1024. 26408528 10.1124/pr.114.010397 PMC4630566

[43] Solmi M, Pigato G, Kane JM, Correll CU . Treatment of tardive dyskinesia with VMAT‐2 inhibitors: a systematic review and meta‐analysis of randomized controlled trials. Drug Des Devel Ther 2018;12:1215–1238. 10.2147/DDDT.S133205 PMC5958944 29795977

[44] Fernandez HH, Stamler D, Davis MD , et al. Long‐term safety and efficacy of deutetrabenazine for the treatment of tardive dyskinesia. J Neurol Neurosurg Psychiatry 2019;90(12):1317–1323. 31296586 10.1136/jnnp-2018-319918 PMC6902058

[45] Josiassen R, Kane JM, Liang GS, Burke J, O'Brien CF . Long‐term safety and tolerability of valbenazine (NBI‐98854) in subjects with tardive dyskinesia and a diagnosis of schizophrenia or mood disorder. Psychopharmacol Bull 2017;47(3):61–68. 28839341 10.64719/pb.4365 PMC5546552

[46] Claassen DO, Carroll B, De Boer LM , et al. Indirect tolerability comparison of deutetrabenazine and tetrabenazine for huntington disease. J Clin Mov Disord 2017;4:3. 28265459 10.1186/s40734-017-0051-5 PMC5331691

[47] Bashir HH , Jankovic J . Treatment of tardive dyskinesia. Neurol Clin 2020;38(2):379–396. 32279716 10.1016/j.ncl.2020.01.004

[48] Bergman H , Soares‐Weiser K . Anticholinergic medication for antipsychotic‐induced tardive dyskinesia. Cochrane Database Syst Rev 2018;1:CD000204. 29341071 10.1002/14651858.CD000204.pub2 PMC6491293

[49] Soares‐Weiser K , Fernandez HH . Tardive dyskinesia. Semin Neurol 2007;27(2):159–169. 17390261 10.1055/s-2007-971169

[50] Rodrigues B , Patil PG , Chou KL . Thalamic deep brain stimulation for drug‐induced tremor. Parkinsonism Relat Disord 2015;21(11):1369–1370. 26350120 10.1016/j.parkreldis.2015.08.033

[51] Whiting BB , Whiting AC , Whiting DM . Thalamic deep brain stimulation. Prog Neurol Surg 2018;33:198–206. 29332084 10.1159/000481104

[52] Buhmann C, Huckhagel T, Engel K , et al. Adverse events in deep brain stimulation: a retrospective long‐term analysis of neurological, psychiatric and other occurrences. PLoS One 2017;12(7):e0178984. 28678830 10.1371/journal.pone.0178984 PMC5497949

