# Supplementary material for: Medical and Surgical Treatment for Medication‐Induced Tremor: Case Report and Systematic Review
Source: Mov Disord Clin Pract. 2022 May 24;9(5):676–87. doi: 10.1002/mdc3.13463 (PMC9274355; doi:10.1002/mdc3.13463)
Supplement: Supplementary file 1 — Appendix S1 Supporting Information [file MDC3-9-676-s001.zip › MDC3_13463_Supplementary file 1.pdf]

Medical and surgical treatment for medication-induced tremor: case report and systematic review

Authors: W.E. Amerika, MD[1], S. van der Gaag,[1] Msc, A. Mosch, MD[1], N.A. van der Gaag, MD PhD[2], C.F.E. Hoffmann, MD PhD[2], R. Zutt, MD PhD[1], J. Marinus, PhD[3], M.F. Contarino, MD PhD[1,3]*

Affiliations:

[1]*Department of Neurology, Haga Teaching Hospital, The Hague, The Netherlands*

[2]*Department of Neurosurgery, Haga Teaching Hospital, The Hague, The Netherlands*

[3]*Department of Neurology, Leiden University Medical Center, Leiden, the Netherlands*

SUPPLEMENTARY TABLE 1: *List of tremorgenic drugs per category, modified from [1] with permission*

**Category**	**Tremorgenic drugs**
Antiarrhythmics	Amiodarone, mexiletine, procainamide
Antibiotics, antivirals and antimycotics	Vidarabine, cotrimoxazole, amphotericin B
Antidepressants and mood stabilizers	Amitriptyline, lithium, SSRIs
Antiepileptics	Valproic acid
Bronchodilators	Salbutamol, salmeterol
Chemotherapeutics	Tamoxifen, cytarabine, ifosfamide, thalidomide
Drugs of abuse	Cocaine, ethanol, MDMA, nicotine, ethanol, MPTP
Gastrointestinal drugs	Metoclopramide, cimetidine
Hormones	Thyroxine, calcitonin, medroxyprogesterone, epinephrine
Immunosuppressants	Tacrolimus, ciclosporin, interferon-alfa
Methylxanthines	Theophylline, caffeine
Neuroleptics and dopamine depleters	Haloperidol, thioridazine, cinnarizine, reserpine, tetrabenazine

SUPPLEMENTARY TABLE 2: *Risk of bias assessment, modified from Marinus et al., 2018. * Studies with > 12 points were classified as good quality, studies with 8-12 points were classified as medium quality and studies with < 8 points were classified as poor quality.*

**No.**	**Criteria**	**Requirements**	**Score***
1	Study objectives	0: The objectives are not clearly stated.<br>1: The objectives are clearly stated.	
2	Study design	0: The study design is not clearly stated.<br>1: The study design is clearly stated.	
3	Characteristics of studied population	0: The studied population’s characteristics are not clearly described.<br>1: Age, disease duration and disease severity are clearly described.<br>2: Age, disease duration and disease severity on a tremor rating scale are clearly described.	
4	Characteristics of non-responders, excluded ones or responders with missing data	0: The characteristics of the non-responders or excluded ones are not described.<br>1: Gender and age of the non-responders or excluded ones are described.<br>2: Gender, age and disease duration of the non-responders or excluded ones are described.	
5	Sampling method for recruitment of study population	0: The sampling method for recruitment of the study population is not appropriately described.<br>1: The location(s) and the type of institution where study population is recruited are mentioned.<br>2: The location(s), type of institution, and number of approached individuals are mentioned.	
6	Sample size	0: 1 patient.<br>1: 2-10 patients.<br>2: > 10 patients.	
7	Choice of the instrument to assess concerned domain justified	0: No justification of the choice of the instrument is given.<br>1: The choice of instrument is justified or the instrument is validated to tremor.<br>2: The choice of instrument is justified and validated to assess tremor.	
8	Type of measurement	0: There is only anecdotal evidence.<br>1: There is at least one subjective measurement.<br>2: There is at least one objective measurement.	
9	Comprehensible statistical methods	0: The statistical methods applied are not fully described.<br>1: The statistical methods applied are fully described.	
10	Agreement/disagreement with other studies	0: Agreements of findings from previous studies are not clearly described.<br>1: Agreements or discrepancies of findings from previous studies are clearly described.<br>2: Agreements and discrepancies of findings from previous studies are clearly described.	
11	Strengths/limitations	0: The limitations of the study are not clearly described.<br>1: The strengths or limitations of the study are clearly described.<br>2: The strengths and limitations of the study are clearly described.	
12	Length of follow-up	0: The study has an unknown duration of follow-up or a follow-up duration less than 1 week.<br>1: The study has a follow-up duration of > 1 week, but ≤ 3 months.<br>2: The study has a follow-up duration of > 3 months.	

SUPPLEMENTARY TABLE 3: *Summary of included level III evidence studies. Treatment effect was determined according to the criteria in the section 'Outcome Measures'. Whenever three or more drugs where implicated in the induction of tardive tremor, the term 'multi-drug' was used.*

**Treatment mg/day**	**Effect***	**Study (year)**	**Design**	**N; age**	**Drug**	**Test**	**Result**	**Comments**
PPNL 0.1 kg^{-1}	Ex	Abila (1985)[33]	DB RCT	6; 22-29	ISPL	QT: Acc	Tremor power '6,5' to '1'.	Tremor power data extracted from Fig 2D.
PPNL 30-80 vs placebo	Mo	Kirk (1973)[3]	SB RCT	10; Unk	LTM	QT: TRS	10 periods '4' or '3' to '2'; 3 periods '4' or '3' to '1'; 7 periods equal.	Significant patient preference for PPNL vs placebo.
PPNL 40	N	Kellett (1975)[4]	SB RCT	15; Unk	LTM	QT: TRS	Less tremor with placebo.	-
ATNL 0.125 kg^{-1}	Mo	Abila (1985)[33]	DB RCT	6; 22-29	ISPL	QT: Acc	'7,5' to 4,5'.	Tremor power data extracted from Fig 2D.
PTL 120	N	Kellett (1975)[4]	SB RCT	15; Unk	LTM	QT: TRS	Less tremor with placebo.	-
STL 0.5 kg^{-1}	Ex	Abila (1985)[33]	DB RCT	6; 22-29	ISPL	QT: Acc	Tremor power '5' to '1'.	Tremor power data extracted from Fig 2D.

List of abbreviations. Acc = Accelerometer; ATNL = Atenolol; DB = Double-Blind; Ex = Excellent; ISPL = Isoprenaline; LTM = Lithium; MD = Multi-drug; Mo = Moderate; N = None; PPNL = Propranolol; PTL = Practolol; QL: Qualitative; QT: Quantitative; SB = Single-Blind; STL = Sotalol; TRS = Tremor rating score; Unk = Unknown. (*) Effect described in study or resulting after calculation (see text for details).

SUPPLEMENTARY TABLE 4: *Summary of included level IV evidence studies sorted by treatment. Treatment effect was determined according to the criteria in the section 'Outcome Measures'. Whenever three or more drugs where implicated in the induction of tardive tremor, the term 'multi-drug' was used.*

**Treatment Mg/day**	**Effect***	**Study (year)**	**Design**	**N, age**	**Drug**	**Test**	**Result (tremor)**	**Comments (# patients)**
PPNL 30-40	Ex	Lapierre (1976)[5]	CS	5; 30-48	LTM	QL: Pe	3x complete control, 2x control	-
PPNL 2 mg i.v.	Ex	Bateman (1981)[6]	CS	2; 46-72	CMTD; CMTD, MTCM	QL: Pe	2x resolution	Case exclusion due to other diagnosis (1).
PPNL 20	Ex	North (1982)[7]	CR	1; 50	LTM	QL: Pe, Hw	Sharply reduced	-
PPNL 40-100	Ex	Karas (1983)[8]	CS, OL	8; Unk	VPT	QL: Pe	8x excellent control	Insufficient accelerometer data available for analysis, therefore test type converted to 'Pe'. Result not mentioned in PPNL treatment (2).
PPNL 18-80	Ex	Zubenko (1984)[9]	CS, OL	4; Unk	LTM	QT: TRS	1x '5' to '2'; 1x '5' to '0'; 1x '4' to '1'; 1x '2' to '0'.	-
PPNL 40	G	Kronfol (1983)[10]	CR	1; 61	IMPM	QL: Sd	Markedly reduced	-
PPNL unk dosage	Mo	Gaby (1983)[11]	CS, OL	1; 26	LTM	QL: Pe QT: Acc	Pe: Reduction	-

PPNL 40	Mo	Lee (2015)[12]	CS	1; 51	MD	QL: Pe	Partially effective	-
PPNL 240	Mi	Stacy (1992)[13]	CS, OL	1; 62	CPMZ	QL: Pe	Little to no change	-
TTBZ 75	Ex	Delecluse (1998)[14]	CR	1; 55	MD	QL: Pe	Remission	TTBZ stopped because of depression (1).
TTBZ 75-187.5	Ex	Shprecher (2012)[15]	CR	1; 74	MTCM	QL: Pe	Remission	-
TTBZ 25	G	Storey (1997)[16]	CR	1; 63	Unk	QL: Pe	Good effect	-
TTBZ 37.5-75	G	Stacy (1992)[13]	CS, OL	5; 34-76	PPNZ/AMTL; CPMZ; MD; MTCM	QL: Pe	1x improvement, 3x marked improvement, 1x almost complete control	-
TTBZ 25-150	**TS** Mi **AIMS** Mi **CGIC** Mi **Avg** Mi	Kertesz (2015)[17]	CS, OL	9; 60-76	DRBA	QT: TRS, AIMS, CGIC	**TS** 1x ‘4’ to ‘1’; 1x ‘2’ to ‘1’; 1x ‘4’ to ‘4’; 3x ‘3’ to ‘3’; 3x ‘2’ to ‘2’. **AIMS** 1x ‘34’ to ‘9’; 1x ‘10’ to ‘4’; 1x ‘14’ to ‘13’; 1x ‘21’ to ‘21’; 1x ‘16’ to ‘16’; 1x ‘12’ to ‘12’; 1x ‘11’ to ‘11’; 1x ‘9’ to ‘9’; 1x ‘23’ to ’27’. **CGIC (‘4’ equals no change)** 3x ‘1’; 2x ‘3’; 1x ‘4’; 2x ‘6’. 1x ‘7’.	Study completion 6 weeks (5/9): non-compliance (2), parkinsonian side-effects (3).
MTPL 100-400	Ex	Zubenko (1984)[9]	CS, OL	4; Unk	LTM	QT: TRS	1x ‘5’ to ‘2’; 1x ‘5’ to ‘0’; 1x ‘4’ to ‘1’; 1x ‘2’ to ‘0’.	Discontinuation MTPL due to wheezing (1) and postural hypotension (2).

MTPL 50-100	G	Gaby (1983)[11]	CS, OL	2; 26-42	LTM	QL: Pe<br>QT: Acc	Pe: well controlled<br>Acc: 50% improvement	No accelerometer data available for MTPL (1; case #1), therefore test type converted to 'Pe'.
MTPL 100	Mi	North (1982)[7]	CR	1; 50	LTM	QL: Pe, Hw	Little improvement	-
MTPL 100	N	Tarsy (2002)[18]	CR	1; 68	MTCM	QL: Pe	No effect	-
BTP 2	Mi	Karas (1983)[8]	CS, OL	2; Unk	VPT	QL: Pe	2x slight reduction	Insufficient accelerometer data available for analysis, therefore test type converted to 'Pe'.
BTP 2	N	North (1982)[7]	CR	1; 50	LTM	QL: Pe, Hw	Failure to resolve	-
BTP 1	N	Tarsy (2002)[18]	CR	1; 68	MTCM	QL: Pe	No effect	-
DPHM 100	Mi	Karas (1983)[8]	CS, OL	2; Unk	VPT	QL: Pe	2x slight reduction	Insufficient accelerometer data available for analysis, therefore test type converted to 'Pe'.
DPHM 200	N	North (1982)[7]	CR	1; 50	LTM	QL: Pe, Hw	Failure to resolve	-
DPHM 100	N	Tarsy (2002)[18]	CR	1; 68	MTCM	QL: Pe	No effect	-
ATNL 50	G	Davé (1989)[19]	CR	1; 45	LTM	QL: Pe	Clear improvement	-
BMCT 7.5	Mi	Stacy (1992)[13]	CS, OL	1; 62	CPMZ	QL: Pe	Little to no change	-
BMCT 2.5	N	Tarsy (2002)[18]	CR	1; 68	MTCM	QL: Pe	No effect	-
CNZP 1.0-	Mi	Patterson	CR	1;	TMTP-SFMT	QL: Pe	Marginal benefit	-

1.5		(1999)[20]		29				
CNZP 0.75	N	Tarsy (2002)[18]	CR	1; 68	MTCM	QL: Pe	No effect	-
INST 3,000	G	Bersudsky (1992)[21]	CS, OL	3; Unk	LTM	QL: Pe	2x complete remission, 1x some improvement	Discrepancy amount patients mentioned in result section (3) with table (2).
INST 8,000	N	Souza (1991)[22]	CS, SB	11; Unk	LTM; MD	QL: Pe	No effect	-
LA 2,500-5,000	Ex	Lieb (1980)[23]	CS	5; 24-71	LTM	QL: Pe	5x complete resolution	Exclusion (1; case #6) due to other diagnosis.
LA 4,000	N	Schou (1980)[24]	CS, OL	6; Unk	LTM	QL: Pe, Sd	1x inconsistent reduction intensity, 5x no change intensity	Inconsistent improvement (1), worst result used for analysis.
NDL 40	Ex	Dave (1994)[25]	CR	1; 25	LTM	QL: Pe	Complete remission	-
NDL 20-80	Mo	Kruse (1984)[26]	CS, OL	6; 21-53	LTM	QL: Pe	2x marked improvement, 3x moderate improvement, 1x slight improvement	-
OPNL 160	**Freq** Mi **Ampl** Mo **Avg** Mo	Brosteanu (1977)[27]	DB RCT	5; Unk	LTM	QT: Acc	**Freq** 15% reduction. **Ampl** 40% reduction.	Preliminary results of trial. High frequency values accelerometer is an artefact. Patients with parkinsonian tremor excluded (5).
OPNL 160-240	1-20) Mo 21-40) Mi **Avg** Mo	Pöldinger (1978)[28]	CS, OL	40; 18-54	1-20) LTM 21-40) NL	QL: Pe	1-20) 11x good response, 4x moderate response, 5x no response 21-40) 4x good response, 5x moderate response, 11x no response	-

PMD 62.5	Ex	Goumentouk (1989)[29]	CR	1; 57	AMTL, LTM	QL: Pe, Hw, Sd, Fnt	Dramatic improvement	-
PMD 750	Mi	Stacy (1992)[13]	CS, OL	1; 48	PPNZ/ AMTL	QL: Pe	Minimal improvement	-
ACTZ 8-14 kg^{-1}	Ex	Lancman (1994)[30]	CR	1; 57	PNT, VPT	QL: Pe	Significant improvement	Tremor started immediately after VPT treatment (1).
AMTD 200	Mo	Karas (1983)[8]	CS, OL	3; Unk	VPT	QL: Pe	3x moderately effective	Insufficient accelerometer data available for analysis, therefore test type converted to ‘Pe’.<br>Patient withdrawn from AMTD therapy because suspected increase in seizures (1).
ARTN 10	Ex	Lee (2015)[12]	CS	3; 51-72	MD; MTZP; DLXT	QL: Pe	1x almost complete remission, 1x substantial reduction, 1x remission	-
C/L 12.5/50	N	Tarsy (2002)[18]	CR	1; 68	MTCM	QL: Pe	No effect	-
CZP 75	Ex	Delecluse (1998)[14]	CR	1; 55	MD	QL: Pe	Remission	-
CPHD unk dosage	Mi	Karas (1983)[8]	CS, OL	4; Unk	VPT	QL: Pe	4x modest relief	Insufficient accelerometer data available for analysis, therefore test type converted to ‘Pe’.
DNPZ 5-10	Ex	Bergman (2005)[31]	CS, OL	6; 64-79	MD	QT: SCII, SAS	**SCII**<br>6x ‘3’ to ‘1’.<br>**SAS**<br>88.9% improvement.	Withdrawal after 4th week due to side effects (1): headache, hypersalivation and nausea.

ETPZ 150	Mi	Stacy (1992)[13]	CS, OL	1; 62	CPMZ	QL: Pe	Little to no change	-
HLPD 6-8	Mi	Tarsy (2002)[18]	CR	1; 68	MTCM	QL: Pe	Mild improvement	-
MTZP 15	N	Tarsy (2002)[18]	CR	1; 68	MTCM	QL: Pe	No effect	-
PTS 16 mEq	Ex	Cummings (1988)[32]	CR	1; 31	LTM	QL: Pe	Resolution	-
PGBL 300	G	Marks (2008)[33]	CR	1; 65	LTM	QL: Pe	Marked improvement	During treatment, lorazepam 1 mg/day changed to clorazepate 30 mg/day (1).
RSP 0.2	N	Tarsy (2002)[18]	CR	1; 68	MTCM	QL: Pe	No effect	-
THXP 6	N	Tarsy (2002)[18]	CR	1; 68	MTCM	QL: Pe	No effect	-
VB6 800-1,200	G	Miodownik (2002)[34]	CS, OL	5; 20-39	LTM	QT: SCII, SAS	**SCII (‘3’ = no change)**<br>1x ‘0’; 3x ‘1’; 1x ‘3’.<br>**SAS**<br>2x ‘3’ to ‘0’; 2x ‘2’ to ‘0’; 1x ‘3’ to ‘2’.	-
Vop-DBS	Mo	Milosevic (2019)[35]	CR	1; 70-79	LTM	QT: TETRAS	‘42’ to ‘29’	-

List of abbreviations. Acc = Accelerometer; ACTZ = Acetazolamide; AIMS = Abnormal Involuntary Motor Scale; Ampl = Amplitude; AMTD = Amantadine; AMTL = Amytriptyline; ARTN = Arotinolol; ATNL = Atenolol; Avg = Average; BMCT = Bromocriptine; BTP = Benztropine; C/L = Carbidopa/levodopa; CGIC = Clinical Global Impression Assessment; CMTD = Cemetidine; CNZP = Clonazepam; CPHD = Cyproheptadine; CPMZ = Chlorpromazine; CR = Case Report; CS = Case Series; CT = Controlled Trial; CZP = Clozapine; DB = Double-Blind; DLXT = Duloxetine; DNPZ = Donepezil; DPHM = Diphenhydramine; DRBA = Dopamine-Receptor Blocking Agent; ETPZ = Ethopropazine; Ex = Excellent; Fnt = Finger-nose testing; Freq = Frequency; G = Good; HLPD = Haloperidol; Hw = Handwriting; IMPM = Imipramine;

INST = Inositol; LA = Linoleic Acid; LTM = Lithium; MD = Multi-drug; Mi = Minimal; Mo = Moderate; MTCM = Metoclopramide; MTPL = Metoprolol; MTZP = Mirtazapine; N = None; NDL = Nadolol; NL = Neuroleptics; OL = Open Label; OPNL = Oxprenolol; Pe = Patient or examiner experience; PGBL = Pregabalin; PMD = Primidone; PNT = Phenytoine; PPNL = Propranolol; PPNZ = Perphenazine; PTL = Practolol; PTS = Potassium; QL: Qualitative; QT: Quantitative; RSP = Reserpine; SAS = Simpson-Angus Scale; SB = Single-Blind; SCII = Subjective Clinical Improvement Impression Scale; Sd = Spiral drawing; TETRAS = The Essential Tremor Rating Assessment Scale; THXP = Trihexyphenidyl; TMTP-SFMT = Trimethoprim-sulfamethoxazole; TRS = Tremor Rating Score; TS = Tremor Scale; TTBZ = Tetrabenazine; Unk = Unknown; VB6 = Vitamin B6; Vop-DBS = Deep brain stimulation in thalamic ventral oral posterior nucleus; VPT = Valproate. (*) Effect described in study or resulting after calculation (see text for details).

SUPPLEMENTARY TABLE 5: *Summary of risk of bias analysis. Studies with > 12 points were classified as good quality, studies with 8-12 points were classified as medium quality and studies with < 8 points were classified as poor quality.*

Author	Year	Total score
**Abila**	1985	**7**
**Bateman**	1981	**7**
**Bergman**	2005	**14**
**Bersudsky**	1992	**4**
**Brosteanu**	1977	**9**
**Cummings**	1988	**4**
**Davé**	1989	**3**
**Dave**	1994	**2**
**Delecluse**	1998	**4**
**Gaby**	1983	**8**
**Goumentouk**	1989	**5**
**Karas**	1983	**7**
**Kellett**	1975	**11**
**Kertesz**	2015	**10**
**Kirk**	1973	**5**
**Kronfol**	1983	**7**
**Kruse**	1984	**7**
**Lancman**	1994	**4**
**Lapierre**	1976	**6**
**Lee**	2015	**7**
**Lieb**	1980	**5**
**Marks**	2008	**4**
**Milosevic**	2019	**10**
**Miodownik**	2002	**11**
**North**	1982	**5**
**Patterson**	1999	**4**
**Pöldinger**	1978	**3**
**Schou**	1980	**6**
**Shprecher**	2012	**6**
**Souza**	1991	**8**
**Stacy**	1992	**8**
**Storey**	1997	**6**
**Tarsy**	2002	**6**
**Zubenko**	1984	**7**

References

1. Morgan, J.C. and K.D. Sethi, *Drug-induced tremors.* Lancet Neurol, 2005. **4**(12): p. 866-76.
2. Abila, B., et al., *The tremorolytic action of beta-adrenoceptor blockers in essential, physiological and isoprenaline-induced tremor is mediated by beta-adrenoceptors located in a deep peripheral compartment.* Br J Clin Pharmacol, 1985. **20**(4): p. 369-76.
3. Kirk, L., P. Baastrup, and M. Schou, *Letter: propranolol treatment of lithium-induced tremor.* Lancet, 1973; 2: 1086–1087.
4. Kellett, J.M., et al., *Beta blockade in lithium tremor.* J Neurol Neurosurg Psychiatry, 1975; 38: 719–721.
5. Lapierre, Y.D., *Control of lithium tremor with propranolol.* Can Med Assoc J, 1976; 114: 619–20, 624.
6. Bateman, D.N., et al., *Cimetidine induced postural and action tremor.* J Neurol Neurosurg Psychiatry, 1981. **44**(1): p. 94.
7. North, D.S. and J.L. Roerig, *Ineffectiveness of metoprolol in controlling lithium-induced tremor.* Clin Pharmacy, 1984; 3: 299-301.
8. Karas, B.J., et al., *Treatment of valproate tremors.* Neurology, 1983; 33: 1380–82.
9. Zubenko, G.S., B.M. Cohen, and J.F. Lipinski, Jr., *Comparison of metoprolol and propranolol in the treatment of lithium tremor.* Psychiatry Res, 1984. **11**(2): p. 163-4.
10. Kronfol, Z., J.F. Greden, and A.P. Zis, *Imipramine-induced tremor: effects of a beta-adrenergic blocking agent.* J Clin Psychiatry, 1983; 44: 225–26.
11. Gaby, N.S., D.S. Lefkowitz, and J.R. Israel, *Treatment of lithium tremor with metoprolol.* Am J Psychiatry, 1983; 140: 593–595.
12. Lee, D.B., Y.S. Woo, and W.M. Bahk, *Use of Arotinolol Pharmacotherapy to Treat Drug-induced Tremor: A Report of Three Cases.* Pharmacopsychiatry, 2015. **48**(4-5): p. 176-8.
13. Stacy, M. and J. Jankovic, *Tardive tremor.* Mov Disord, 1992. **7**(1): p. 53-7.
14. Delecluse, F., J.A. Elosegi, and J.M. Gerard, *A case of tardive tremor successfully treated with clozapine.* Mov Disord, 1998. **13**(5): p. 846-7.
15. Shprecher, D., *Sensory trick with metoclopramide-associated tardive tremor.* BMJ Case Rep, 2012. **2012**.
16. Storey, E. and J. Lloyd, *Tardive tremor.* Mov Disord, 1997. **12**(5): p. 808-10.
17. Kertesz, D.P., et al., *Tetrabenazine for tardive tremor in elderly adults: a prospective follow-up study.* Clin Neuropharmacol, 2015. **38**(1): p. 23-5.
18. Tarsy, D. and G. Indorf, *Tardive tremor due to metoclopramide.* Mov Disord, 2002. **17**(3): p. 620-1.
19. M., D., *Treatment of lithium induced tremor with atenolol.* Can J Psychiatry, 1989; 34: 132–133.
20. Patterson, R.G. and R.L. Couchenour, *Trimethoprim-sulfamethoxazole-induced tremor in an immunocompetent patients.* Pharmacotherapy, 1999. **19**(12): p. 1456-8.
21. Bersudsky, Y., et al., *The effect of inositol on lithium-induced polyuria—polydipsia in rats and humans.* Human Psychopharmacology: Clinical & Experimental, 1992: p. 403-407.
22. Souza, F.G.M., et al., *The effects of lithium discontinuation and the non-effect of oral inositol upon thyroid hormones and cortisol in patients with bipolar affective disorder.* J Affect Disord, 1991; 22: 165-170.
23. Lieb, J., *Linoleic acid in the treatment of lithium toxicity and familial tremor.* Prostaglandins Med, 1980; 4: 275–279.
24. Schou, M., *Linoleic acid in the treatment of lithium-induced tremor: a pilot trial with negative outcome.* Prostaglandins Med, 1980; 5: 343–344.
25. Dave, M. and M.M. Langbart, *Nadolol for lithium tremor in the presence of liver damage.* Ann Clin Psychiatry, 1994; 6: 51-52.
26. Kruse, J.M., L. Ereshefsky, and M. Scavone, *Treatment of lithium-induced tremor with nadolol.* Clin Pharm, 1984; 3: 299–301.

27. Brosteanu, E.F., L.; Kaiser, H., *Double-blind trial with oxprenolol versus placebo in the treatment of lithium-induced tremor.* In: Kielholz P, ed, Beta-Blockers and the Central Nervous System. Baltimore, MD: University Park Press, 1977.
28. Pöldinger, W., *Therapy of extrapyramidal side effects, with particular reference to persistent dyskinesia and lithium tremor.* Int Pharmacopsychiatry, 1978; 13: 230–233.
29. Goumentouk, A.D., T.A. Hurwitz, and A.P. Zis, *Primidone in drug-induced tremor.* J Clin Psychopharmacol, 1989. **9**(6): p. 451.
30. Lancman, M.E., J.J. Asconape, and F. Walker, *Acetazolamide appears effective in the management of valproate-induced tremor.* Mov Disord, 1994; 9: 369.
31. Bergman, J., et al., *Beneficial effect of donepezil in the treatment of elderly patients with tardive movement disorders.* J Clin Psychiatry, 2005. **66**(1): p. 107-10.
32. Cummings, M.A., K.L. Cummings, and M.G. Haviland, *Use of potassium tot treat lithium's side effects [letter].* Am J Psychiatry, 1988; 145: 895.
33. Marks, D.M., C.U. Pae, and A.A. Patkar, *Potential role of pregabalin in the treatment of lithium-induced tremor: a case report.* Int J Neuropsychopharmacol, 2008; 11: 879–881.
34. Miodownik, C., E. Witztum, and V. Lerner, *Lithium-induced tremor treated with vitamin B6: a preliminary case series.* Int J Psychiatry Med, 2002. **32**(1): p. 103-8.
35. Milosevic, L., et al., *Case Studies in Neuroscience: Lack of inhibitory synaptic plasticity in the substantia nigra pars reticulata of a patient with lithium-induced tremor.* J Neurophysiol, 2019. **122**(4): p. 1367-1372.
