# Supplementary material for: Medical and Surgical Treatment for Medication‐Induced Tremor: Case Report and Systematic Review
Source: Mov Disord Clin Pract. 2022 May 24;9(5):676–87. doi: 10.1002/mdc3.13463 (PMC9274355; doi:10.1002/mdc3.13463)
Supplement: Supplementary file 1 — Appendix S1 Supporting Information [file MDC3-9-676-s001.zip › MDC3_13463_Supplementary file 2.pdf]

Medical and surgical treatment for medication-induced tremor: case report and systematic review

Authors: W.E. Amerika, MD[1], S. van der Gaag[1], MSc, A. Mosch, MD[1], N.A. van der Gaag, MD PhD[2], C.F.E. Hoffmann, MD PhD[2], R. Zutt, MD PhD[1], J. Marinus, PhD[3], M.F. Contarino, MD PhD[1,3]*

Affiliations:

[1]Department of Neurology, Haga Teaching Hospital, The Hague, The Netherlands

[2]Department of Neurosurgery, Haga Teaching Hospital, The Hague, The Netherlands

[3]Department of Neurology, Leiden University Medical Center, Leiden, the Netherlands

Supplementary file 1: Search string performed 10-12-2019

Pubmed (Legacy version:
"tardive tremor"[tiab] OR "medication-induced tremor"[tiab] OR "drug-induced tremor"[tiab] OR "drug induced tremor"[tiab]

Embase:
"tardive tremor” OR "medication-induced tremor" OR "drug-induced tremor" OR "drug induced tremor"

Cochrane Library:
"tardive tremor” OR "medication-induced tremor" OR "drug-induced tremor" OR "drug induced tremor"

Limits:
- English Language
- Human
